# Local applications of kerbside food and garden organics collection: An Australian regional study

**DOI:** 10.1177/0734242X261429260

**Published:** 2026-03-30

**Authors:** Christine Blanchard, Peter Harris, Celmara Pocock, Bernadette K McCabe

**Affiliations:** 1 Centre for Agricultural Engineering, University of Southern Queensland, Toowoomba, QLD, Australia; 2 Manager Environment and Resource Recovery, Gympie Regional Council, Gympie, QLD, Australia; 3 End Food Waste Cooperative Research Centre, Urrbrae, SA, Australia; 4 Centre for Heritage and Culture, University of Southern Queensland, Toowoomba, QLD, Australia

**Keywords:** FOGO, landfill diversion, municipal solid waste, willingness to pay, community attitudes, social and behaviour change, small-scale composting

## Abstract

Literature is emerging on Australian household food and garden waste disposal and recycling habits; however, little is known about the feasibility of, and community attitudes to, kerbside organics collection in regional settings. This study provides results on the conversion of food and garden organics to compost in a council-owned small scale, boutique composting system and employed a series of surveys to evaluate household residents’ attitudes to the collection and processing of food organic and garden organic material in two regional towns in the Lockyer Valley Regional Council, Queensland. The trial achieved substantial diversion of organic waste from the general waste stream, which was reduced by 31%. Extrapolation showed that implementation of a similar collection system across the entire council region would extend the current landfill life by at least 2 years. The composting system cost less than 200,000 Australian dollars (AUD) to establish and eliminated considerable transport and gate fee costs for commercial composting, which would have exceeded 500,000 AUD for the treatment of the tonnes of food waste collected in the trial. Pre- and post-trial surveys showed residents’ willingness to pay increased from 44% to 50% and the range of costs the respondents were willing to pay was between 22.80 and 42.80 AUD. Key to improving residents’ attitudes lie in clearly communicating how waste charges are applied and the costs to councils to manage waste. Further recommendations to enhance the overall trial effectiveness include sufficient lead in time for trial preparation, strategic communication and recycling education specifically related to separation of food.

## Introduction

Food waste is a global concern, with the large amounts of food directed to landfill creating considerable financial and environmental costs. Globally, around 1.3 billion tonnes of food waste is generated annually, costing the global economy around 940 billion United States dollars (USD) each year and emitting around 9 gigatonnes of carbon dioxide equivalent (CO_2_e) emissions into the atmosphere (New South Wales Environmental Protection Authority, 2022; Rohini et al., 2020). Australian households dispose of more than 3 million tonnes of edible food waste every year, costing the average Australian home 2200 Australian dollars (AUD; 1 USD ≈0.66 AUD) annually and emitting an estimated national total of 6.6 million tonnes of CO_2_e (Australian Government, 2017). Landfilling organic municipal solid waste consumes valuable landfill space and contributes to climate change through the release of greenhouse gases including methane and CO_2_. The global warming potential of methane is 87 times more than CO_2_ when considering its impact over 20 years (International Energy Agency, 2023; Parameshwari, 2017). It is therefore imperative, and in keeping with the food recovery hierarchy, that this organic material be recovered as animal feed, or converted to energy, soil conditioners and compost (Ceryes et al., 2021; Commonwealth of Australia, 2020; Soma, 2022; United States Environmental Protection Agency, 2022).

Kerbside collection of household waste in most of the developed world is carried out by local governments using collection systems supported by waste management policy initiatives that seek to change community behaviour (Chowdhury, 2009; Knickmeyer, 2020; Pérez et al., 2021). In Australia, garden organics or food organics and garden organics (FOGO) collection is emerging, and different drivers shape how councils pursue recovery of organics. These include state legislation mandating such collections, limited or no access to cost-effective landfill, and government policy drivers to improve environmental outcomes by diverting organic waste from landfill (Edwards et al., 2018). While these drivers encourage or mandate the adoption of FOGO services by councils, councils must also consider the ability of the local community to support and pay for such services. Householders need to understand the rationale for FOGO and actively contribute to its implementation by managing an additional kerbside stream for the separation of organic waste. While this is essential given the financial and environmental costs of the large quantity of food waste created by households (Edwards et al., 2018; Hu et al., 2021; Wakefield and Axon, 2020), it can prove challenging. A literature review, conducted by Blanchard et al. (2023), identified the cost of organics collection and householders’ reluctance to pay for FOGO services, and a lack of regulation and inconsistent legislation across Australian jurisdictions as major barriers. However, if these barriers can be addressed, councils could see improved outcomes in management of organic waste and increased uptake of FOGO collection at the household level.

In Australia, local councils manage 10 million tonnes of general waste collected from kerbside bin services every year (Australian Local Government Association, 2022). This waste is generally landfilled or sent for resource recovery (Australian Local Government Association, 2021). Kerbside-collected waste is typically 40–50% FOGO that could be recovered and converted to compost to improve local soils (Commonwealth of Australia, 2020; Lockyer Valley Regional Council, 2019; Queensland Government, 2023). Diversion of FOGO from landfill is therefore highly desirable. Australia has 537 local councils, which vary in population size across metropolitan, regional, rural and remote areas. Local councils have access to different levels of resource recovery options depending on location and waste stream. Policy levers driving change in waste management come from national and state tiers of government and remain in their infancy compared with European waste management, which includes landfill diversion as standard practice (Blanchard et al., 2023).

Processing of organic waste is dependent on a range of factors including logistics and facility costs, which provide unique challenges in regional Australia (Gillespie and Halog, 2023; Refsgaard and Magnussen, 2009; Youssef et al., 2022). Regional councils are required to meet the same standards as urban councils but face several challenges including limited or no access to organic processing facilities, long transport distances to commercial composting markets, lower council rates as a revenue base from which to fund waste management, and smaller waste volumes which lack an economy of scale for large-scale composting or anaerobic digestion treatment. In many cases, support mechanisms intended to assist regional councils in overcoming these logistical, locational and funding-related barriers are either lacking or inaccessible. Further challenges include insufficient funding and workforce capacity, contamination of the FOGO stream and the resulting compost product, limited opportunities for collaboration and knowledge sharing among regional stakeholders and the absence of standardized messaging to facilitate community education and engagement (Landells et al., 2024, 2025).

At the same time, metropolitan councils face their own distinct set of structural constraints, including servicing multi-unit developments, long-term locked-in collection contracts, limited availability of industrial land for processing facilities and strong community resistance to the siting or expansion of waste infrastructure. Comparative research by Wall (2020) demonstrates that both metropolitan and regional councils in New South Wales encounter substantial, albeit different, institutional, spatial, contractual and social challenges when advancing FOGO systems. Accordingly, the capacity of councils to implement organics recovery is shaped not by location alone, but by context-specific governance, infrastructure, market and community conditions.

Recent evidence further demonstrates that regional councils are not pursuing a single pathway towards FOGO implementation, but rather a diversity of institutional and geographic strategies. In New South Wales, regional local governments have in many cases advanced FOGO adoption more rapidly than metropolitan local governments, employing a range of governance models including cross-council partnerships, shared processing facilities and regional clustering of feedstock to achieve economies of scale and manage costs (Palisi et al., 2025). These collaborative regional approaches complement locally managed council-owned models and highlight the importance of context-specific institutional design in shaping viable organics recovery systems across non-metropolitan Australia.

While Australian studies have examined kerbside organics collection and community attitudes in regional settings (Ames and Cook, 2020; Wall, 2020; Williams et al., 2024), there remains a gap in the literature regarding the viability of decentralized and localized treatment models where local governments collect FOGO and undertake composting on council-owned land for direct civic use. The location of regional councils, relative to commercial composting operations, can therefore present an opportunity for locally managed treatment options. In many urban councils where FOGO systems have been introduced, proximity of commercial composters allow councils to send collected material to a third party for processing. This third party assumes responsibility for the material and its end-use market. Where such a third-party processor is not readily available economically, a council must consider other options or not introduce the service. In this context, using boutique or bespoke composting options to manage organic material close to source makes sense in a triple bottom line context and warrants further study. Small-scale composting processes such as aerated static pile systems provide a low-cost system suitable for regional councils where organic waste diversion is affordable, provided there are not considerable transport costs. In traditional urban composting treatment, commercial composters rely on customers purchasing the material for industrial or commercial use. This can burden the composter and can see inflated gate fees for the council to manage the risk with finding appropriate markets for this material. When treating the material locally, there are additional options available to the council for the use of the compost product including parks, gardens, community tree plantings, community gardens and other relevant uses, which can benefit the local community.

There is an emerging body of evidence on Australian household food waste disposal and recycling habits (Nguyen et al., 2022); however, little is known about the feasibility of, and community attitudes to, kerbside organics collection in regional settings. This research aimed to first assess the viability of kerbside FOGO collection in a Queensland regional council area by drawing on results from a trial of FOGO conversion to compost in a council-owned small-scale system. Second, the study evaluated community attitudes to kerbside FOGO collection by understanding residents’ attitudes and willingness to use and pay for a service.

## Background of FOGO trial

### Location of case study

**Figure 1.**
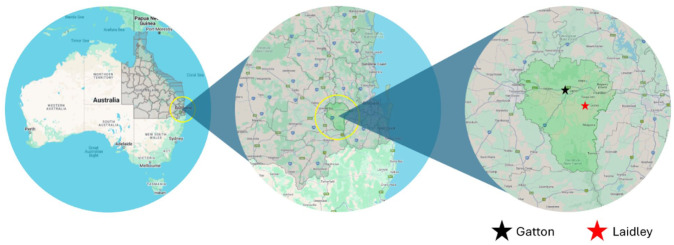
Location of FOGO trial council in Queensland, Australia, adapted from Queensland Government (2025). FOGO: food organics and garden organics.

Lockyer Valley Regional Council area has a population of almost 43,000 and is located 90 km from Queensland’s capital city, Brisbane, in Southeast Queensland (Figure 1). The two case study towns, Gatton and Laidley, are the largest towns in the Lockyer Valley region. Located 20 km apart, both towns are serviced by schools, supermarkets, banking, post office and hardware. Waste management in both towns comprises kerbside waste collection and drop off waste facilities. The FOGO trial area was selected for convenience of waste truck servicing, which reduced costs by only including households that formed part of a single waste truck run. It was also the only local council to undertake onsite processing of organics in Queensland.

### Pre-trial general waste characteristics

The Lockyer Valley Regional Council undertook a waste characterization audit before the trial commenced to enable council to make informed decisions about waste management options. Organic material contributed 40% of the general kerbside waste stream: 25.24% was organic material and 14.77% was garden waste, both suitable for a FOGO system (see Supplemental Material 6.7 – Section 4.1.1; Envirocom Australia, 2019). The high percentage of organic waste in the stream indicated that a kerbside collection system for organic waste was viable and led to the development of the FOGO collection system trial, which was implemented by council for 1 year.

### Description of kerbside collection

Prior to the trial, waste collection services included a weekly 240 litre general waste (red lid) bin collection and a two-weekly 240 litre recycling (yellow lid) bin collection. During the FOGO trial, 1021 households (477 in Laidley and 544 in Gatton) were provided with a weekly 240-litre FOGO bin service, a 7-litre kitchen benchtop caddy, and a roll of 150 compostable liners. One month before commencing the service, a letter was mailed to the residents outlining the new service and the timeframe, and bins, caddies and liners were delivered. A package of literature was supplied with the bins including advice on what to put in each bin and when the bins would be collected (Supplemental Material 6.1). Participants retained their 240-litre general waste bin, but general waste moved to a two-weekly service schedule. The two-weekly 240-litre recycling bin service remained unchanged (Lockyer Valley Regional Council, 2022a). The service was automatically supplied to all residents in the trial area; however, residents could elect not to use the FOGO bin (Lockyer Valley Regional Council, 2024).

## Methodology

### Survey design, administration and response rate

Council surveyed residents in the trial area pre- and post-trial to determine attitudes and experiences of residents in the FOGO trial. Pre-trial surveys were administered in August 2021 and post-trial surveys in August 2022. Survey questions (Supplemental Material 6.2 and 6.3) were determined by the programme funder, Queensland State Government, and were grouped into seven main categories for both the pre- and post-trial surveys including demographics, attention paid to waste management, consideration of environmental benefits of FOGO recycling, importance of the FOGO trial, use of the FOGO bin, willingness to pay for the FOGO service and general comments (Supplemental Material 6.4). Surveys were administered in hard copy form mailed to residents, with return via reply-paid envelope. Pre-trial (*n* = 1021) and post-trial (*n* = 1021) surveys were administered to each household in the trial. Response rates of 20% (201) and 19% (194) were achieved for the pre- and post-trial surveys, respectively, which is consistent with expectations for voluntary mail-based surveys but introduces potential for non-response bias. Survey results were analysed in consideration of the opportunities, challenges and barriers for council to provide a FOGO service (Ramshaw, 2024).

### Demographic characteristics of participants

The trial area demographic was diverse, including social housing, culturally and linguistically diverse, National Disability Insurance Scheme participants and those with varied medical needs. Pre-trial respondents primarily resided in Laidley (107), with 81 living in Gatton, and 13 blank responses. There were similar numbers post-trial with 101 residing in Laidley and 93 in Gatton. Respondents were primarily older single or couple households, with limited numbers of families; 77% of respondents lived in a one to two people household, 13% lived with three to four people household, and 7% lived with five or more people. Of these households, 24% were under 20 years, 20% were 21–40 years, 22% were 41–60 years, and 68% of respondents were aged over 61 years. Australian Bureau of Statistics data were consistent with that found in the trial (Supplemental Table S2).

At the commencement of the trial, some residents with special needs contacted council, and appropriate processes were established to ensure these residents could participate in the FOGO trial if they chose. These included, but were not limited to, provision of additional general waste bins to manage the high volumes of household waste and provision of additional caddy liners. While households with limited English proficiency were not formally identified as a discrete cohort with the trial design, residents who self-identified or raised communication challenges with council staff were provided additional support and tailored graphics to ensure they understood the types of materials to be recycled in each bin.

### Waste stream audits

Domestic kerbside waste streams were audited by an external company at trial start, mid and end of the trial to determine waste stream change throughout the trial. The first audit was conducted 2 weeks before the trial commenced. General waste was collected from all 1021 households, with sub-samples representative of the equivalent mass of 75 bins drawn from each sample. Materials were examined as presented for disposal and hand-sorted by the audit team. Each sample was deposited in a safe and clean sorting area, and materials were categorized according to the established standard ASTM D5231 – 92 (Reapproved 2016) Standard Test Method for Determination of the Composition of Unprocessed Municipal Solid Waste (ASTM International, 2016). Sub-categories were included to identify food/kitchen as FOGO acceptable, and either containerized, containerized in a compostable bag or non-containerized. Several additional Queensland Department of Environment and Science and Innovation categories of interest were also included, specifically disposable nappies and food/kitchen waste in compostable or non-compostable packaging.

Materials were sorted and weighed to the nearest 0.01 kg to determine composition. An additional assessment was conducted on food waste items to further understand the types of food waste found within the stream. A subsample was drawn from each food waste category by a wheeled loader at the end of the assessment day and coned-and-quartered to achieve a manageable sorting size while maintaining representativeness. Food waste materials were then sorted into established categories.

### Small-scale trial conversion of FOGO to compost

Collected organic material was composted using an aerated static pile system (Supplemental Material 6.6). This system required minimal establishment costs and little operational labour as the composting material does not require turning nor generate nuisance odour.

### Analytical approach, statistical analysis and modelling

This study employed a mixed-methods analytical approach comprising three complementary components: analysis of survey data, analysis of waste audit data and qualitative analysis of free-text responses.

Survey data were summarized using counts and proportions to describe resident attitudes, behaviours and service perceptions before and after the trial. Changes between the pre- and post-trial surveys were examined at the overall group level, as individual households could not be matched across the two survey periods.

Qualitative free-text survey responses were analysed using inductive thematic analysis, with themes developed iteratively through repeated reading and coding of responses.

Waste audit data were analysed by calculating changes in waste composition and diversion rates across audit periods, supported by yield and tonnage measurements to assess system performance over the trial period.

Statistical analysis was conducted on continuous data and categorical data. Continuous data were analysed for differences using ANOVA and, where sample sizes were below 20, Kruskal-Wallis tests were used. Pairwise Tukey HSD was used to determine significance between pairs. Categorical data were dummy encoded into binary variables (0’s and 1’s) for further analysis. Chi-squared testing was used to identify relationships, and standardized residuals were determined for reporting under- and over-representation within groups and post hoc residual analysis for significance. An alpha value of 0.05 was used for all statistical tests. Consequently, all occurrences of the term ‘significant’ refer to instances where the *p*-value was <0.05.

Modelling was conducted to explore seasonality within the continuous data (Supplemental Material 6.9). The least squared method was used in conjunction with the Microsoft Excel Solver function to fit a sine wave curve with *x*- and *y*-axis offsets to data (Equation 1).

Equation 1: Sine wave formula

$$y=A\sin\left(k\left(t-x_{\mathrm{offset}}\right)\right)+y_{\mathrm{offset}}$$

where *A* is the amplitude, *k* is the wave number, *t* is the time, *x*_offset_ and *y*_offset_ enable the fit to move left and right, and up and down, respectively, on the cartesian plane.

### Limitations of study

A limitation of this study is that the demographic composition of respondents does not align with the typical Australian population, particularly in terms of age distribution and household sizes (Supplemental Table S2). The trial area was primarily older single or couple households, with limited numbers of families. Waste generation in this demographic is lower than those with families, and this may have contributed to a lower proportion and tonnages of food waste being placed in bins as this demographic is not traditionally wasteful (Karunasena et al., 2021). Per capita, single-person households waste, by far, the most food because food is not packaged for single-person consumption, and younger single-person household residents have lifestyles attuned to irregular patterns of eating at home (Baker et al., 2009; Pearson et al., 2013). Ideally, the sample would more closely reflect the national demographic profile to enhance the generalizability of the findings, but it was not possible to locate such an area in the Lockyer Valley with the required number of waste services that was able to be serviced in the same truck run.

A further limitation is the study’s reliance on survey-based data to interpret household motivations, values and behavioural drivers. Although household visits were undertaken to provide operational support where required, the study did not incorporate in-depth qualitative interviews capable of more fully capturing the social dynamics shaping participation.

Although the dataset includes quantitative measures collected before and after the trial, the analysis was intentionally framed as exploratory and evaluative rather than inferential. Pre- and post-trial survey respondents could not be reliably matched at the household level, response rates were modest, and the trial was designed as a programme evaluation. Under these conditions, formal hypothesis testing risks overstating precision and implying causal relationships that the data cannot support. Accordingly, observed changes in survey measures should be interpreted as indicative trends that inform understanding of community response rather than as statistically validated effects.

Overall, these factors indicate that the findings should be interpreted as context-specific and exploratory. The respondent sample is likely to over-represent the perspectives of households for whom participation in FOGO services is comparatively easier, and conclusions regarding behavioural change, service acceptance and willingness to pay should therefore not be generalized beyond the trial context.

## Results and discussion

### FOGO collection data and contamination

Presentation of FOGO bins for weekly collection was low compared with general waste and recycling as a baseline. While weekly general waste and two-weekly recycling bins were presented 93% and 78% of the time, respectively, the FOGO bins averaged only 52% ± 6.9% weekly. FOGO bin presentation rate was seasonally affected with presentation rates peaking in summer around 62% and falling to around 42% in the winter (Supplemental Figure S3). This was expected as green waste typically increases substantially during the warmer months, while cooler temperatures in the winter reduce food waste decay and associated ‘yuck factor’. While householders were not asked how often they presented their bins for collection, 85.2% of householders used the bin at least once during the trial period, 27% used the bin more than 75% of the time, 12% used the bin more than 90% of the time, and 2.8% used the bin 100% of the time. Anecdotally, bins from single-person or older person households were not presented unless they were full to avoid ‘wasting a driver’s time to empty them’, supporting the idea that this was not less use of the bin, rather less frequent presentation (L. Giles, personal communication, 2021).

**Figure 2.**
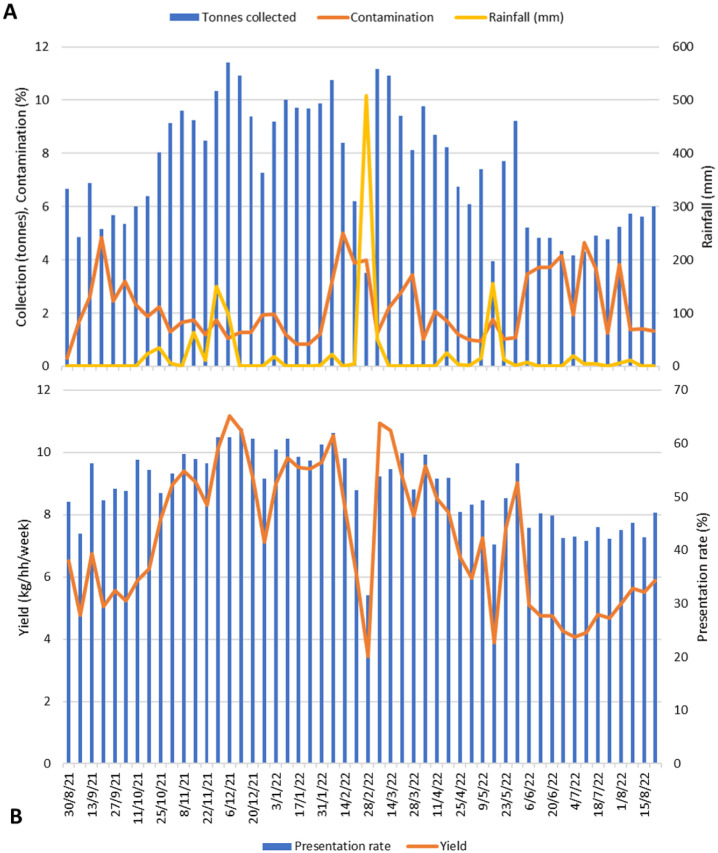
FOGO collection data showing (a) tonnes (Mg) collection versus rainfall versus contamination, and (b) presentation rate versus yield per household. FOGO: food organics and garden organics.

The quantity of FOGO collected during the trial indicates that households who engaged with the service were diverting a substantial volume of organic material, demonstrating the capacity of the service to achieve higher diversion under broader participation. Collection tonnages also varied seasonally ranging from around 11 tonnes collected weekly in the summer, and around 4 tonnes collected weekly in the winter (Supplemental Figure S4). During the trial, 386 tonnes of FOGO were collected with an average yield of 7.26 ± 2.20 kg per household per week. Although overall presentation rates were low, this yield is comparatively high when benchmarked against reported diversion rates in other Australian regions where as little as 4.5 kg per household was diverted (De Silva and Taylor, 2024; Local Government NSW, 2021). This suggests that frequency of presentation does not necessarily equate to volume of use. Tonnage collected, presentation rate and yield per household all aligned well with seasonal variation expectations (Figure 2(a) and (b)). In February 2022, a natural disaster was declared following an intense period of rainfall (Lockyer Valley Regional Council, 2022b). Collection tonnages remained high for several weeks as the weather remained warm, and large volumes of garden waste and grass clippings were actively managed by householders.

Contamination was generally low, though extreme weather events demonstrated potential for increasing contamination. While contamination rates averaged 2.17% ± 1.19% (Figure 2(a)), contamination peaked at 5% following an extreme rainfall event. Despite this surge, the monthly average contamination rate was not significantly impacted. Residents understood the FOGO bin was for garden waste, but the food component of the waste stream required further education. The pantry/fridge cleanout was a primary source of contamination from containerized food waste placed incorrectly in either the FOGO bin or the general waste bin, without first being separated from packaging. Increased and ongoing communications is needed to encourage residents to transfer food from packaging into caddy liners or directly into the FOGO bin. This presents an opportunity to improve the quantity and quality of food waste removed from the general waste stream and ultimately diverted to organic recovery.

Additionally, understanding of the tools used to market products is poor, and this drives additional contamination. Such tools include labelling of biodegradable, compostable or non-compostable and are subjected to greenwashing, where companies apply green marketing strategies to gain competitive advantage (Allison et al., 2021; Kakadellis et al., 2021; Szabo and Webster, 2021). Information was provided to residents to seek additional liners from council if needed or to purchase liners themselves but only those liners compliant with Australian Standard AS4736 Commercial Composting should be used in the FOGO system. However, there was evidence in the separated contamination that incorrect liners were used: either plastic bags or non-compliant liners. This could also be addressed in communication with residents on how to manage contamination and requires a more comprehensive behaviour change programme. For example, Etim et al. (2025) showed that the theory of planned behaviour (TPB) can positively influence food waste behaviour.

#### Impact of FOGO collection on general waste stream composition

During the trial, the relative percentage of acceptable food and garden waste in the general waste bins decreased from 27.28% to 18.08% (Supplemental Material 6.7); however, potentially compostable waste increased from 3.29% to 21.63%. The principal compostable material lost to general waste included fine garden waste, containerized food waste, cardboard, disposable paper products and acceptable food waste. The main food items lost to general waste included bread (usually still packaged in plastic bags) and bones. Householders may need longer than the 12-month period of this trial to change habitual behaviours and for recycling of organics to become normalized (Thomas and Sharp, 2013).

Values in Supplemental Material 6.7 can be used to estimate the organics diversion that might be achieved across the general waste stream if FOGO were rolled out across the entire Council area. In 2021–2022, Council collected 10,287 tonnes of general waste from the kerbside stream. At 31% organics recovery from the general waste bin, a whole-of-council rollout is expected to divert 3200 tonnes of FOGO from landfill annually, and an additional 2000 tonnes of FOGO could be recovered with further efforts to improve resident behaviours.

Interestingly, the FOGO trial appears to have had additional benefits for the recyclable materials. Total recyclables within the general waste bin reduced from 13.67% to 9.7% over the duration of the trial (Supplemental Material 6.7). Albeit only a change of 4 percentage points, this represents an additional recovery of around 400 tonnes of recyclables otherwise destined for landfill. The precise drivers of this improvement cannot be determined from the available data. However, the change may reflect broader behavioural shifts associated with heightened engagement during the trial period, including increased exposure to council communications and greater attention to correct material separation (Miliute-Plepiene et al., 2016). It is also possible that some households became more mindful of separating recyclable packaging from food waste as part of their participation in the FOGO service. Nevertheless, ongoing contamination of FOGO bins by containerized food indicates that these behavioural changes were uneven and that separation practices remained imperfect.

While these results indicate a meaningful behavioural change during the trial period, the survey findings that inform interpretation of service acceptance and engagement must be understood within the demographic context of the sample. The response rate was approximately 20%, and respondents were predominantly older residents living in single- or two-person households. These characteristics are known to influence waste generation behaviour, service engagement and willingness to pay and therefore limit the extent to which the findings can be generalized to the broader community.

The age-based patterns identified in this study reinforce this limitation. Younger households were significantly more likely to report capacity constraints, difficulty managing the service and concerns regarding pests and odour, while older households were significantly more likely to report that they did not find the service difficult and expressed a need for additional information about FOGO. Respondents aged 41–60 were significantly more likely to indicate low interest in the service. These demographic effects indicate that behavioural responses to FOGO are strongly shaped by life stage and household composition and that the observed levels of acceptance and behavioural change in this survey cannot be assumed to apply uniformly across the wider population.

Accordingly, the results should be understood as context-specific and exploratory, reflecting the particular demographic structure of the trial community rather than providing a definitive representation of broader regional behaviour. Nevertheless, the findings offer valuable insight into how different household groups experience and respond to FOGO services, supporting the need for targeted engagement strategies rather than for uniform programme design.

### Community attitudes to kerbside FOGO collection

Free-text comments in the post-trial surveys were subject to thematic analysis (Supplemental Material 6.8). Themes emerging from the data are presented in the following sub-sections and provide insight into residents’ experience of the service, perceived value and factors influencing participation and willingness to pay.

There were age-based differences in perceptions of the FOGO service. Younger respondents (21–40 years) were significantly more likely to report household capacity constraints and difficulty managing the service, while older respondents (61+ years) were significantly more likely to report that they did not find the service difficult. These findings indicate that acceptance and perceived ease of use vary systematically across demographic groups, with household composition and life-stage influencing service experience.

Concern regarding pests and odour emerged as a dominant issue among younger respondents, particularly those aged 21–40, both with and without children (*p* < 0.05). In contrast, no strong demographic association was observed for claims of insufficient waste generation or general resistance to the service (‘I just don’t want to’). These results demonstrate that participation barriers are not uniform across the community but are shaped by life stage and household composition.

#### Frequency of collection

**Figure 3.**
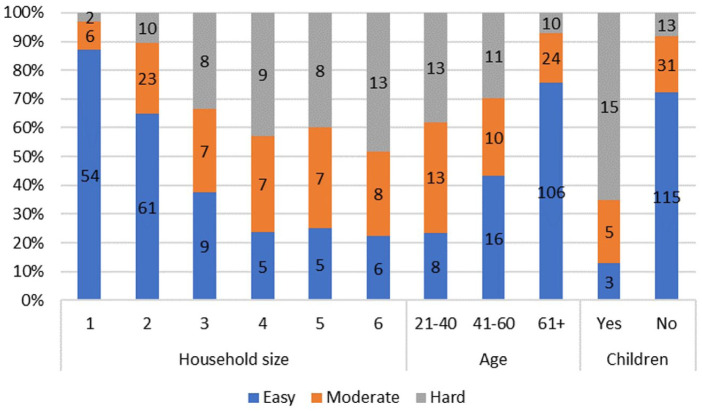
Effect of household size, resident age and presence of children on general waste management difficulty.

Additional themes, including perceived necessity of the service, interest in the provision of free compost and views on service costs, emerged in qualitative responses and are reported descriptively. A minority of respondents had concerns regarding frequency of general waste collection and ability to separate waste into the different recycling categories. While 70% of residents reported having no issues managing their general waste, 16% of free-text feedback indicated that these respondents disliked general waste collection being reduced from weekly to two-weekly. Controlling for age group and the presence or absence of children in the household identified that the 41–60 age group without children were having substantial problems with general waste bin capacity and two-weekly collection (*p* < 0.05). By comparison, once children were present in a household, all age groups cited a large family as a difficulty in using the system (*p* < 0.05) but, interestingly, did not indicate that general waste capacity or two-weekly collection were major issues. Typically, single persons and older ages in households manage with the given general waste capacity every 2 weeks and separated their waste into the correct bins (*p* < 0.05; Figure 3). This is consistent with the understanding that smaller households of older peoples tend to generate less waste and may have more time to consider and implement changes to waste management and behaviours. When residents expressed concerns about management of general waste to council, staff offered support to help residents understand their waste stream and ensure the correct bins were being used. Very few residents accepted this offer, suggesting the problem was perhaps not as acute as residents implied.

Households with more than two people and including children indicated difficulties managing waste during the trial (*p* < 0.05). Childless households became over-represented when three or more adults were present, becoming over-represented in fortnightly collection of general waste and large family groups (*p* < 0.05). Interestingly, in households with children present, large family was not over-represented until six people present in the home. Also of interest, three-person households with children citing difficulty with general waste capacity and fortnightly collection were over-represented (*p* < 0.05), but all other household sizes with children were represented as expected. Around 70% of households with young children (19%) experienced difficulty managing two-weekly general waste collection. While the survey did not identify types of waste that might create issues for households, anecdotally, disposable nappies would likely have contributed substantially (L. Giles, personal communication, 2021). Changing general waste to two-weekly servicing creates space and odour issues for households with disposable nappies and sanitary products. This could be resolved with an opt-in nappy service where residents can secure an extra weekly general waste service to dispose of odorous nappies and personal hygiene products, an option that was successfully adopted by a New South Wales local council (Penrith City Council, 2024).

#### Participants’ attitudes to willingness to use and pay

Willingness to pay for waste services remains a challenge for councils that are subjected to increasing pressure to take responsibility for finding sustainable solutions for excessive amounts of solid waste (Akhtar et al., 2017). Pre-trial, willingness to pay was 44% while, post-trial, willingness to pay was 50% (Table 1). Within the willing group, the field-weighted average cost the respondents were willing to pay was AUD 32.8 ranging from 22.8 to 42.8 (Table 2). While this was a positive outcome, a large portion of the surveyed population remained unwilling to pay for the service, and how to convert residents into willing payers is a critical question to increase uptake. By comparison, in Central Queensland, a region similar to the trial area, 58% of residents were willing to pay for a domestic food waste diversion option but the amount they were willing to pay was unlikely to cover the service costs (Benyam et al., 2020). For context, at the time of the trial, as society was emerging from the COVID-19 pandemic, so too was the cost-of-living crisis emerging. International research indicated consumers have been more willing to pay for sustainable products since the pandemic (Dangelico et al., 2022). However, the Lockyer Valley region was also heavily impacted by major floods in early 2022 and was still recovering when surveys were sent to householders. Consequently, households were under considerable financial stress following the impacts of floods and a cost-of-living crisis, and willingness to pay for new services may have been negatively impacted. This sentiment was reinforced in the free-text responses in which 6% cited: already high costs of rates; residents should not have to pay to ‘recycle’; or the cost of living was currently too high and such a service adds a burden to already financially compromised households.

**Table 1.** Pre-trial willingness to pay.

Willingness to pay (*n* = 199)^a^
Strongly supportive (25%)
Supportive (19%)
Neutral (22%)
Opposed (9%)
Strongly opposed (25%)

a Percentages are based on eligible responses only, whereby renters and non-responses were excluded.

**Table 2.** Post-trial willingness to pay.

Amount to pay (*n* = 151)^a^
AUD 60–80 (1%)
AUD 40–60 (9%)
AUD 20–40 (36%)
AUD <20 (4%)
AUD 0 (50%)

AUD: Australian Dollars.

a Percentages are based on eligible responses only, whereby renters and non-responses were excluded.

Education is a key factor to drive community uptake and willingness to pay. Pricing of services has direct implications for constituent welfare and economic well-being, and there is ample opportunity to improve community understanding of how council prices community services. This is demonstrated by free-text commentary, suggesting ‘Council should provide the service at no additional charge’. Influence on pricing is exerted by a broad range of factors; however, councils are vague and lack explanation on how pricing is developed (Carnegie and West, 2010). To the constituent, waste management is funded through their council rates, and the cost of waste management is often not transparent, leading to a false assumption that waste management/recycling services are free (Alzamora and Barros, 2020). In the case of this council, the cost to collect and transport the FOGO waste, provision of the bin, caddy and liners, the education and communication programme, the composting system and other associated charges are all expenses incurred by council that must be recouped through a waste management charge.

Council must also raise awareness of critical issues facing the community. One such issue is the rapid depletion of available landfill airspace. A key consequence of this is the anticipated increase in waste management costs, driven by the need to transport residual waste to third-party landfills located outside the region. Although this challenge is outlined in council’s publicly available waste strategy (Lockyer Valley Regional Council, 2019), there has been limited public communication regarding the severity of the issue and its potential impact on future waste charges. Greater transparency and proactive engagement around these strategic pressures may enhance community understanding of the rationale for the FOGO trial and foster stronger support for council’s broader waste diversion objectives. Analysis of attitudinal responses revealed important age-based differences in engagement with FOGO. Respondents aged 21–40 were significantly more likely to indicate that they were not interested in FOGO, while respondents aged 41–60 were significantly more likely to report that they required additional information to understand the importance of the service. No strong age-based differences were observed in perceptions regarding landfill capacity constraints or the role of a circular economy, suggesting that these broader sustainability concepts are recognized relatively consistently across age groups. These findings indicate that engagement with FOGO is shaped by both informational needs and motivational factors that vary by life stage.

#### Barriers to the use of the service

Engaging community to utilize compost product may garner increased buy-in. Respondents expressed interest in compost being used in council park and gardens (52%); made available to the community to purchase (37%); used in community gardens (35%); used for rehabilitation of local land (31%); used in local schools and early learning centres (24%); used for rehabilitation of landfill (22%); made available for local community groups (17%); and sold to farmers at low cost (1%). Council advised residents that collected organic material would be processed, with the resulting compost intended for civic applications such as community tree planting, restoration of flood-damaged waterways and other locally relevant environmental initiatives. Making this product available to residents and the community may allow residents to feel they are engaging with the programme and receiving value in return for their efforts.

**Figure 4.**
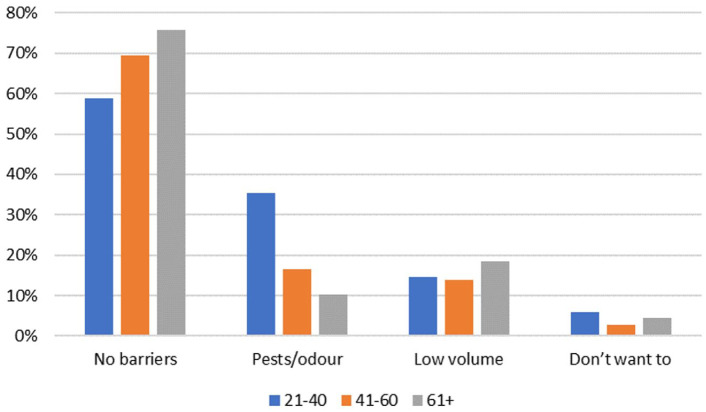
Barriers to kerbside FOGO adoption for various age groups. Note: totals add to more than 100% due to respondents providing more than one barrier response. FOGO: food organics and garden organics.

There were few barriers to using the new service and participation in the FOGO trial was high (Figure 4). Nevertheless, some persistent barriers to uptake remained regarding hygiene and education. Despite householders’ intent to separate food waste, they also held concerns about FOGO bins leading to pests and odour (*p* < 0.05). This is concerning, as Oehmann et al. (2022) reported that the distinct ‘yuck factor’ often undermined the behavioural intent of participants, especially where individuals have no prior experience in separating food waste. About 18% of respondents were concerned about pests and odour. Respondents in the 21–40 age group in particular noted pests and odours as potential barriers, possibly related to the management of disposable nappies as previously discussed (*p* < 0.05).

Prior research indicates that household roles, competing priorities, perceptions of odour and the ‘yuck’ factor, and responsibility for waste separation significantly influence engagement with organics services (Ames and Cook, 2020; Evans, 2011; Oehman et al., 2022). Future research incorporating interviews, home-based observations and participatory methods would provide deeper insight into these dynamics, particularly for larger households and households with children, where participation challenges were most evident in this trial. Education is critical to improving adoption. It was sometimes challenging for residents to translate information from printed educational material into personal understanding and knowledge. For instance, a very small percentage (less than 3%) indicated they simply did not want to use the bin. When council officers communicated with these residents directly, they were usually converted to FOGO users once they understood that it was easy to do (L. Giles, personal communication). Personalized communication to some residents, while time consuming and expensive for council, is essential to ensure buy-in to the service across all residents. While information provision and education emerged as important components of the trial, these findings suggest that impersonal or ‘hands-off’ communication alone is unlikely to be sufficient to drive sustained behavioural change. It is important to distinguish between general communication activities (e.g. flyers, bin stickers, and written materials) and more personalized or immersive forms of engagement that involve direct interaction with households. Personalized engagement enables residents’ specific concerns, anxieties and situational constraints to be addressed, particularly where behavioural change involves discomfort, uncertainty or competing household priorities (Boulet et al., 2023).

Prior research demonstrates that social norms, visceral responses, and feelings of disgust associated with food waste are often stronger drivers of sorting behaviour than abstract knowledge of sustainability benefits (Ames and Cook, 2020; Waitt and Phillips, 2016). Practice-based perspectives similarly emphasize that information alone rarely transforms behaviour without experiential reinforcement that reshapes everyday conventions and routines (Shove, 2010). Comparative research by Wall (2020) further illustrates that the most effective FOGO programmes combined information provision with immersive engagement strategies, including one-on-one interactions between council officers and households, involvement of wider community networks (e.g. supermarkets, landlords and owners’ corporations) and return of compost to residents as a tangible reinforcement of the recycling process. These insights align closely with the results of this study and help explain why direct engagement proved more effective than written communication alone in resolving participation barriers.

Similarly, around 20% of residents believed they did not produce enough food or garden waste to warrant using a FOGO bin. Discussions with these residents helped to allay fears that they would be wasting council’s time by using the bin for only very small amounts of food. Again, this resulted in more residents adopting the FOGO bin system, suggesting that prioritizing education that no amount is too small could increase food waste recovery. Importantly, service information was only provided 1 month prior to commencement; notification at least 3 months prior to service commencement would give residents time to understand the system, query council and begin to change behaviours in how they manage household waste prior to commencement.

Foundational community education regarding the nature and purpose of the FOGO trial is needed. The 21–40 age group were over-represented in the ‘I don’t care about FOGO’ group (*p* < 0.05). Meanwhile, the 41–60 age group were over-represented in indicating they ‘need more information’. Specifically, council needs to more effectively educate these age groups to raise awareness of the critical issue facing council: the rapid depletion of available landfill airspace. A key consequence of this situation is the anticipated increase in waste management costs, driven by the need to transport residual waste to third-party landfills located outside the region. Although this challenge is outlined in council’s publicly available waste strategy (Lockyer Valley Regional Council, 2019), there has been limited public communication regarding the severity of the issue and its potential impact on future waste charges. Greater transparency and proactive engagement around these strategic pressures may enhance community understanding of the rationale for the FOGO trial and fostered stronger support for council’s broader waste diversion objectives.

#### Method of communication

**Figure 5.**
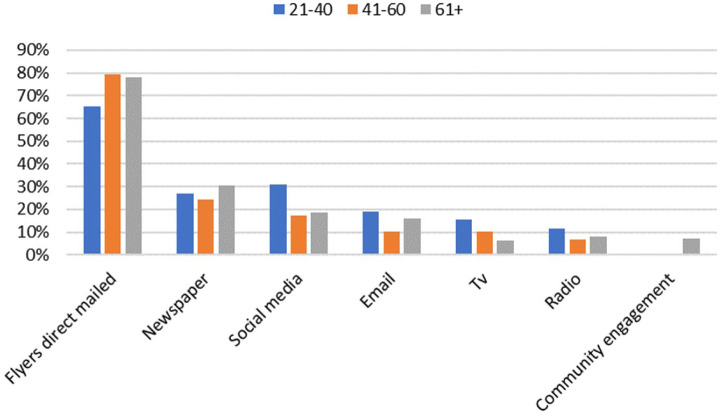
Preferred method of communication with respondents.

Choosing the correct communication channel is essential to ensuring messages are directed appropriately to the community. The preferred method of communication regarding important changes and updates was consistent between age groups (Figure 5). Direct mail flyers were preferred by 74% of residents, followed by newspapers at 27%, suggesting residents like physical information that could possibly be retained for future reference or that such documents have an authoritative nature compared with other contemporary styles of communication. There is no substantial literature on methods councils use to communicate with residents, and this warrants further research to better understand how to best communicate information.

The findings of this study intersect with broader economic and institutional debates on municipal waste management, particularly in relation to service pricing, cost recovery and the role of local government in shaping household behaviour. While this study is not positioned as a formal economic or institutional analysis, the results reinforce emerging evidence that governance arrangements, funding constraints and service design play a critical role in determining waste system performance and citizen participation. By providing detailed empirical insights from a regional Australian trial, this research contributes applied evidence to this wider conversation while remaining grounded in the operational realities of local government service delivery.

### Recommendations

There were four main factors that determined the success of the trial: (1) staff engagement with the community, (2) communication, (3) education and (4) local treatment of organics (Table 3). The following recommendations aim to address these areas and enhance the overall effectiveness and uptake of the programme.

**Table 3.** Success factors and recommendations.

Success factor	Recommendations
Staff engagement with community	• Direct staff engagement with resistant community members can substantially improve outcomes • Promote and encourage local champions • Engage local media to capture engaged residents/champions and their success stories
Communication	• Allow sufficient time (i.e. 3+ months) of notice prior to service commencement • Flyers, letters and newsletters are effective communication tools • Local newspaper articles and social media posts should be utilized • Council’s online engagement hub with questions and answers • Community events where the compost is used on council’s parks and gardens • Face-to-face meetings with council staff and/or elected members
Education	• Community service charges should be explained to residents • Critical waste management issues facing the community should be explained to residents • Have staff or online resources readily available to answer questions • Engage collection drivers to report visible contamination • Engage community throughout service period
Local organics treatment	• Phase out weekly residual waste over time • Consider options to compost locally and promote success stories • Community access to end-use compost products • Use compost on local applications • Create jobs in the local area, provide environmentally positive solutions and keep waste/resource recovery close to source to eliminate transport costs

Beyond these operational recommendations, the findings also point to the importance of broader institutional and governance conditions in shaping household participation and service outcomes. While this study primarily engages with technical, behavioural and policy-oriented dimensions of FOGO implementation, issues raised by participants regarding service costs, willingness to pay, transparency of waste charges and the role of council in influencing behaviour reflect challenges that are inherently economic and institutional in nature.

These findings support the need for a more integrated analytical approach to regional FOGO design, in which technical service delivery is examined alongside governance quality, institutional arrangements and funding structures. A system-thinking framework as noted by Iacovidou et al. (2021) and Jha et al. (2024) offers a particularly valuable lens for this purpose, as it enables the interaction between policy settings, financial mechanisms, institutional capacity, community engagement and operational performance to be analysed as a connected system rather than as isolated components. Applying a system-thinking approach to FOGO planning and governance can assist local governments in identifying structural constraints, unintended policy effects and leverage points for reform, thereby strengthening both service performance and long-term community participation and warrants further study.

Consistent with this interpretation, broader empirical evidence further supports the central finding of this study that effective recycling systems are fundamentally shaped by their wider economic and institutional context. International research by Kostakis et al. (2026) consistently shows that higher recycling performance is associated with national wealth, sustained investment in waste management infrastructure, prudent public debt management, strong governance capacity and education levels. These findings reinforce that improvements in recycling outcomes are not driven solely by technological upgrades or regulatory controls, but by the creation of enabling economic and institutional environments in which environmental objectives are aligned with long-term economic planning, human capital development and public service capacity. The results of this regional FOGO trial reflect this same dynamic at the local government scale: service viability and community participation were not determined by any single intervention, but by the degree of alignment across governance structures, funding mechanisms, regulatory settings, operational capacity and community engagement. This system-level alignment helps explain why technical solutions alone are insufficient to deliver durable recycling outcomes, particularly in regional contexts where institutional resources and market conditions are more constrained.

## Conclusion

This study examines a novel model of localized FOGO treatment for regional councils, providing a unique applied context for evaluating a decentralized approach to organics recovery. The trial generated substantial operational benefits, with general waste reduced by 31% and projections, suggesting that full-scale implementation across the local government area may extend the life of the local landfill by approximately 2 years. The use of a cost-effective, small-scale composting system eliminated considerable transport and gate fees associated with commercial processing, while the compost end-product created a valuable local resource for council parks, gardens and community projects. In addition, application of compost to tree plantings contributed to improved soil condition and vegetation resilience in flood-impacted areas of the region, highlighting the broader environmental co-benefits of localized organics treatment.

The findings also contribute applied insight to the emerging literature on household waste behaviour in regional Australia, where empirical evidence on community responses to kerbside FOGO remains limited. While the trial suggests that community engagement played an important role in service uptake, behavioural and economic interpretations should be viewed as indicative and context-specific. Willingness to pay increased from 44% to 50% between the pre- and post-trial surveys and, although there was no relationship identified between age and willingness to pay, there is a substantial demographic skew in the respondent group may not be translatable to the population at large. More targeted engagement strategies may enhance participation and willingness to pay over time, but their long-term impact requires further investigation.

Future research should therefore focus on refining community engagement approaches, exploring the social implications of altered general waste collection frequencies, and examining longer term behavioural trajectories associated with FOGO participation. Additionally, how residents prefer to receive official information from council warrants further exploration. Together, these insights will support the development of resilient, locally appropriate organics recovery systems that balance operational performance with community acceptance and financial sustainability.

## Supplemental Material

sj-docx-1-wmr-10.1177_0734242X261429260 – Supplemental material for Local applications of kerbside food and garden organics collection: An Australian regional study Supplemental material, sj-docx-1-wmr-10.1177_0734242X261429260 for Local applications of kerbside food and garden organics collection: An Australian regional study by Christine Blanchard, Peter Harris, Celmara Pocock and Bernadette K McCabe in Waste Management & Research
