# Supplementary material for: Local applications of kerbside food and garden organics collection: An Australian regional study
Source: Waste Manag Res. 2026 Mar 30;44(8):1170–83. doi: 10.1177/0734242X261429260 (PMC13354837; doi:10.1177/0734242X261429260)
Supplement: sj-docx-1-wmr-10.1177_0734242X261429260 – Supplemental material for Local applications of kerbside food and garden organics collection: An Australian regional study [file sj-docx-1-wmr-10.1177_0734242X261429260.docx]

# Supplementary Materials

## Bin suite and collateral supplied with bins

Figure S1 shows the three bins that form the standard waste service for residents. The lime green lid is the FOGO bin used in the trial discussed in this paper. The kitchen caddy, liners, and literature were all distributed with the FOGO bin.


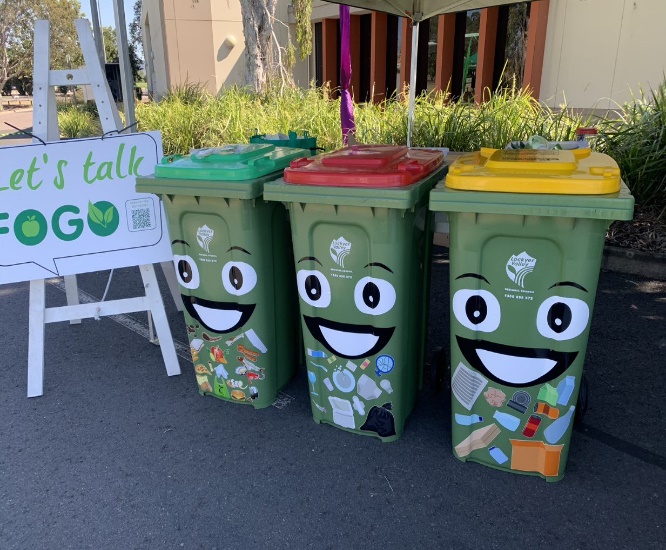

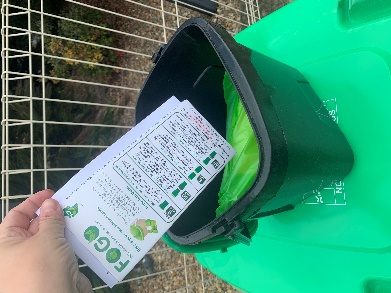

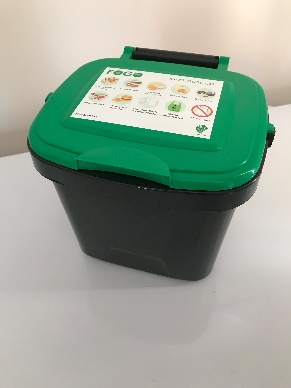


Figure S1: (left to right) FOGO, general waste, and recycling bins, FOGO kitchen caddy, compostable liners and education brochure, and kitchen caddy with stamp. Photographs by Lori Giles.

##
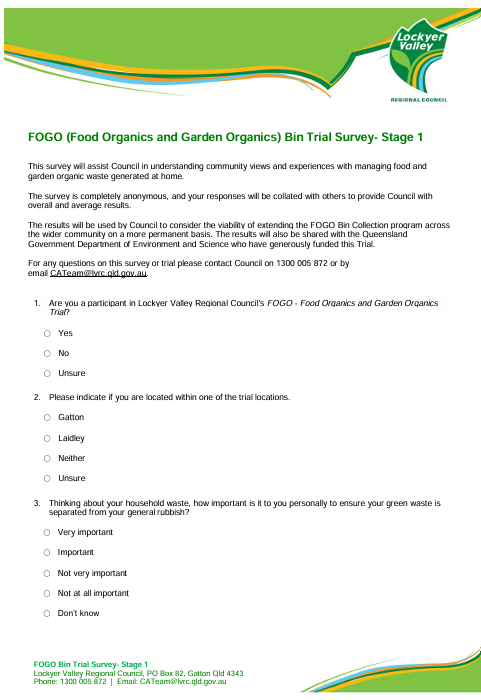
Pre-trial survey


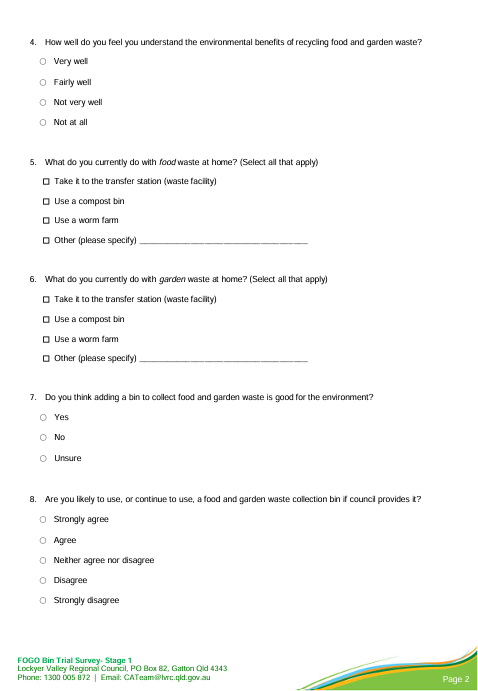


##
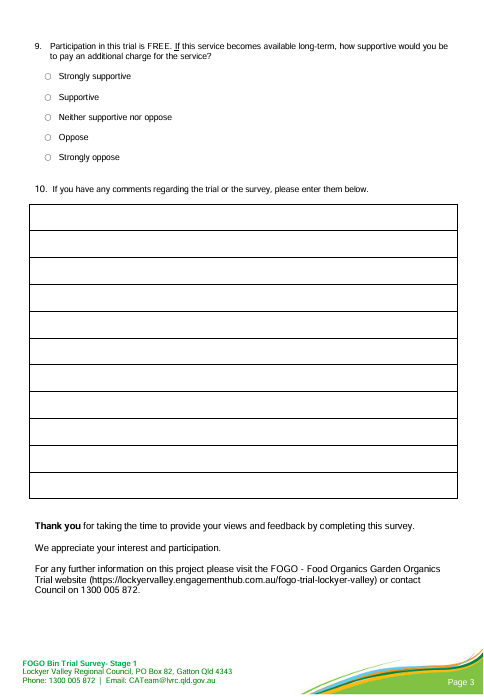
6.3 Post-trial survey


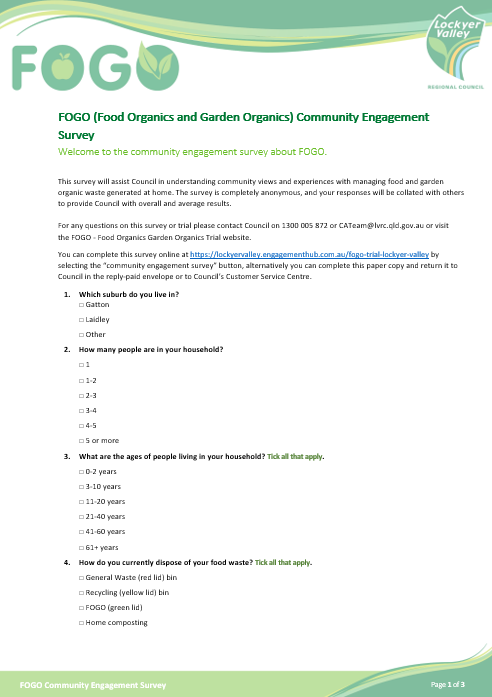


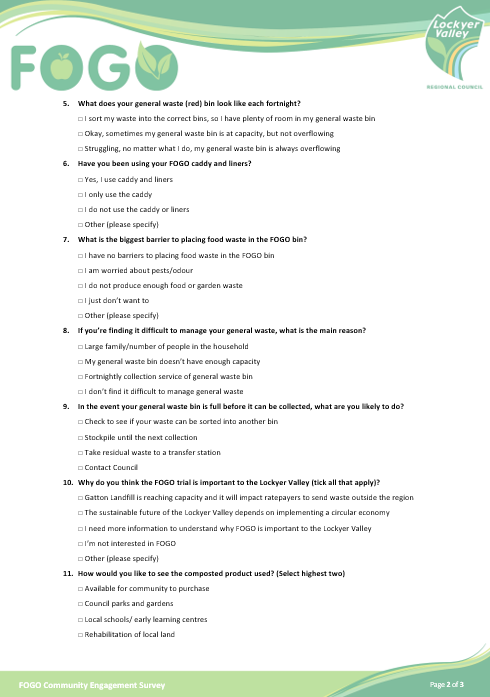


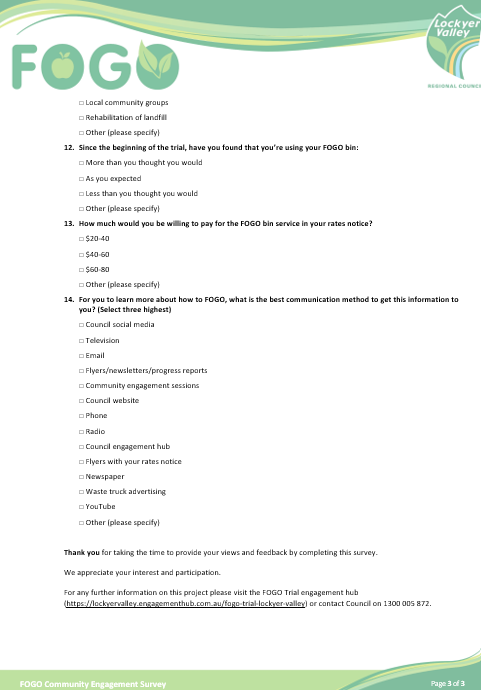


## FOGO Trial Design Survey Questions

Table S1: FOGO trial design survey questions

| Pre -trial | | Post-trial | |
| --- | --- | --- | --- |
| Factor (source) | Question | Factor (source) | Question |
| Demographics | Confirm participation  Locality | Demographics | Locality  Number people in household  Age |
| Attention paid to waste management | Importance of separating green waste  Current disposal of food waste | Attention paid to waste management | Current disposal  General waste bin  Use of caddy and liners |
| Consideration of environmental benefits of FOGO recycling | Understanding of environmental benefits of recovering organic waste  What is currently done with food/garden waste | Concern about barriers to adoption | What is biggest barrier to placing food in FOGO bin  Main reason for difficulties in managing general waste  What is done if general waste bin is full |
| Importance of FOGO trial | Adding a bin to collect FOGO is good for the environment | Importance of FOGO trial | Why the project is important  How should the composted product be used |
| Use of FOGO bin | Likely to use food/garden waste bin | Use of FOGO bin | Is use of bin more or less than expected |
| Willingness to pay for service | Willing to pay for service long term | Willingness to pay for service | How much is reasonable for a FOGO service |
| Comments | General comments | Communication | How Council should best communicate information  General comments |

## Demographic characteristics of participants in trial area

Table S2: Demographic characteristics of participants surveyed compared to 2021 Australian Bureau of Statistics (ABS) census data (.idcommunity, 2023)

|  |  | N% | ABS Census 2021 (%) |
| --- | --- | --- | --- |
| Gatton residents  N = 81 (pre-trial)  N = 100 (post-trial) | Gatton households in trial – 544 | Pre-trial 14.89%  Post-trial 18.38% | Total population 7,851  Total dwellings 3,135  N=17.35% |
| Laidley residents  N = 107 (pre-trial)  N = 111 (post-trial) | Laidley households in trial – 477 | Pre-trial 22.43%  Post-trial 23.27% | Total population 4,402  Total dwellings 1,987  N=24% |
| Number of people in household combined | 1  1-2  2-3  3-4  4-5  5+ | 29%  43%  8%  8%  3%  6% | Couples with children  Couples without children  One parent family  Lone person households  Group households |
| Ages living in household combined | 0-2 years  3-10  11-20  21-40  41-60  61+ | 3%  11%  10%  20%  22%  68% | 0-4 years 651  5-11 957  12-17 853  18-24 1288  25-34 2035  35-49 1951  50-59 1273  60+ 3180 |

Note: ABS data combined for Gatton, Laidley and Laidley North (.idcommunity, 2023)

## Small scale trial conversion of FOGO to compost

The collected organic material was composted using an aerated floor, static pile system. This system required minimal establishment costs, minimal labour to operate as it does not require turning the composting material nor generate odour as a nuisance (Radicle Agriculture, 2023). The process consists of two pipes with aeration holes (Figure S2-A), a fan to blow air through the pipes, and a solar panel and inverter to power the fans (Figure S2-B). During the active phase of the composting process, the organic material remains on the pipes for twelve weeks after which time it is screened to remove contamination (Figure S2-C-F). When the screening is complete, the material is sampled and tested against the parameters in Council’s Environmental Authority Permit to ensure the product is safe for land application (Queensland Government, 2022).


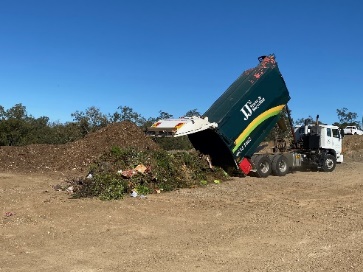

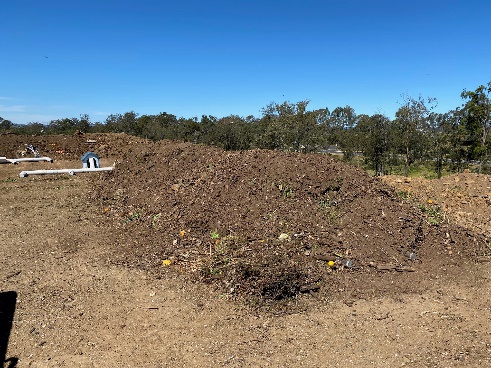

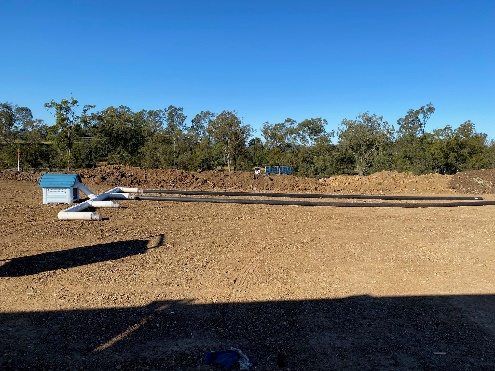

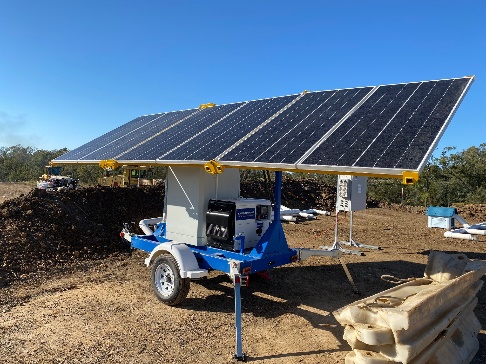

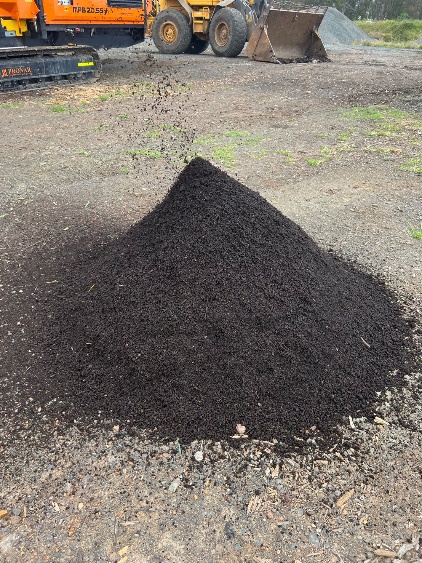

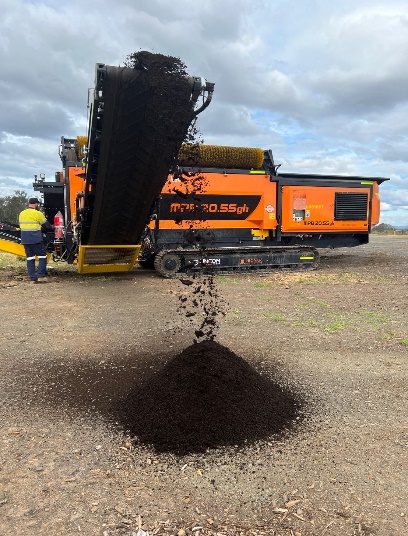


Figure S2: Photographs of compost treatment system: Aeration pipes (A), solar panel and inverter system (B) first load of FOGO delivered (C), first load of FOGO on aeration pipes (D), screening of FOGO (E), and final screened FOGO soil conditioner (F). Photographs by Christine Blanchard.

## Waste stream audit results pre-, mid- and post-trial

Table S3: Waste stream audit results pre-, mid-, and post-trial

| **Material Category** | **Average Proportion (%)** | | | | |
| --- | --- | --- | --- | --- | --- |
|  | **General Waste Bin** | | | **FOGO Bin** | |
|  | **Pre-Trial Audit** | **Mid-Trial Audit** | **Post-Trial Audit** | **Mid-Trial Audit** | **Post-Trial Audit** |
| Recyclable Paper | 7.14 | 0.6 | 0.53 | 0.05 | 0.04 |
| Recyclable Glass | 1.96 | 1.18 | 1.09 | 0.09 | 0.14 |
| Recyclable Plastics | 2.96 | 6.45 | 5.42 | 0.22 | 0.26 |
| Recyclable Metals | 1.61 | 2.6 | 2.66 | 0.13 | 0.13 |
| **Total Recyclable** | **13.67** | **10.83** | **9.7** | **0.49** | **0.57** |
| Non-Recyclable Paper | 4.65 | 7.36 | 10.35 | 0.34 | 0.26 |
| Non-Recyclable Glass | 0.04 | 0.01 | 0.09 | 0.01 | 0.00 |
| Non-Recyclable Plastics | 18.58 | 20.20 | 15.73 | 1.1 | 0.58 |
| Non-Recyclable Metals | 1.81 | 3.32 | 4.08 | 0.11 | 0.02 |
| **Total Non-Recyclable** | **25.08** | **30.89** | **30.25** | **1.56** | **0.86** |
| Household Hazardous | 0.73 | 1.5 | 0.77 | 0.00 | 0.04 |
| Others | 2.08 | 0.79 | 1.21 | 0.40 | 0.00 |
| Other Organic | 5.3 | 7.5 |  | 0.99 | 0.48 |
| Other fine material <12.5 mm | 5.15 | 6.41 | 6.82 | 0.08 | 0.06 |
| **Total Others** | **13.26** | **16.2** | **8.8** | **1.47** | **0.58** |
| Acceptable food/kitchen | 8.33 |  | 5.91 |  | 10.03 |
| Acceptable Garden | 17.15 | 3.87 | 10.19 | 4.95 | 81.52 |
| Acceptable Other Putrescible | 1.8 | 11.41 | 1.98 | 86.52 | 3.17 |
| **Total Acceptable Food and Garden Waste** | **27.28** | **15.28** | **18.08** | **91.47** | **94.72** |
| Unacceptable Food/Kitchen | 14.19 | 0.00 | 8.89 |  | 1.29 |
| Unacceptable Garden |  | 9.61 | 0.33 | 1.37 | 0.99 |
| Unacceptable Other Putrescible | 3.22 |  | 2.32 | 2.17 | 0.26 |
| **Total Unacceptable Food and Garden Waste** | **17.41** | **9.61** | **11.54** | **3.54** | **2.54** |
| Potentially Compostable Paper | 3.29 | 3.01 | 4.36 | 0.19 | 0.17 |
| Potentially Compostable |  | 9.93 |  | 1.23 |  |
| Compostable Paper |  | 0.55 | 8.85 | 0.06 | 0.57 |
| Compostable |  | 3.7 |  |  |  |
| Other Organic |  |  | 8.42 |  |  |
| **Total Potentially Compostable** | **3.29** | **17.19** | **21.63** | **1.48** | **0.74** |

## Summary of commentary from post-trial survey respondents

Table S4: Summary of commentary from post-trial survey respondents

| **Theme** | **Number (*n* = 195)** | **Barrier/Enabler** | **Example Quote** | **Commentary** |
| --- | --- | --- | --- | --- |
| Very happy with the service | 42 | Residents indicated they were happy with the service and wanted it to continue. | I love the FOGO caddy and green bin. Am amazed how much of the kitchen scraps can go into composting and really hope the FOGO becomes a permanent service. Keep up the good work Council. Well done!! | These residents welcomed and embraced the service and found it fitted in with their lifestyles. |
| Would prefer general waste servicing to remain weekly | 31 | Residents struggled with volume of general waste if only collected two-weekly. | We are really struggling with general waste being collected two-weekly. Baby nappies smell really bad. We don't mind the FOGO bin, but it could be the one collected two-weekly not the general waste. | Notes here suggest residents want to embrace the service but have some initial barriers that may be readily overcome. |
| Not willing to pay for the service | 8 | Comments indicated residents were not necessarily against the service but did not want to pay for it. | Maintain the service free of charge. | Cost of living pressures may contribute to this comment and these residents may be happy to embrace the service if there was no charge.  There is little understanding of why councils charge for waste and how these charges are developed and passed on to constituents. |
| Don’t need the service | 7 | Residents indicated they didn’t need the service: either because they already managed their FOGO waste on site, or they generated insufficient FOGO waste to warrant such a service. | FOGO bin not required on acreage. Green waste goes to livestock. Other waste goes to either livestock or worm/compost patch. | A mix of housing types in the trial area showed some residents who already managed their organic waste may have been less interested in the service. |
| Pests and/or odour | 6 | Residents noted they were not happy with the pests and odour generated from the kitchen caddy or the FOGO bin. | The liners ooze out the juice of fruit and veg, resulting in a pool of smelly rotting liquid in the bin, which attracts flies, then of course maggots are climbing out of the bin. Then after you spend time cleaning it, the smell has penetrated the plastic of the bin. The extra work is not ideal for the elderly and the food scrap will just as easily decompose in the red bin. | More communication from Council on how to better manage the food scraps, caddies, and liners may assist in shifting the opinions of these residents. |
| Would like free compost to be provided by Council | 4 | Residents felt they were providing the input material for the composting process, and they should be provided with the final product at no charge as part of the program. | Would be good if the Council had a give - away day for the FOGO soil for resident's gardens once or twice a year, so we benefit from the costs of the bins. | Such a service could be introduced to demonstrate circular economy benefits, that is, separating organic waste into bins leads to the production compost that could be made available at small cost or free of charge. This is an achievable option for Council. |

## Modelling for seasonality


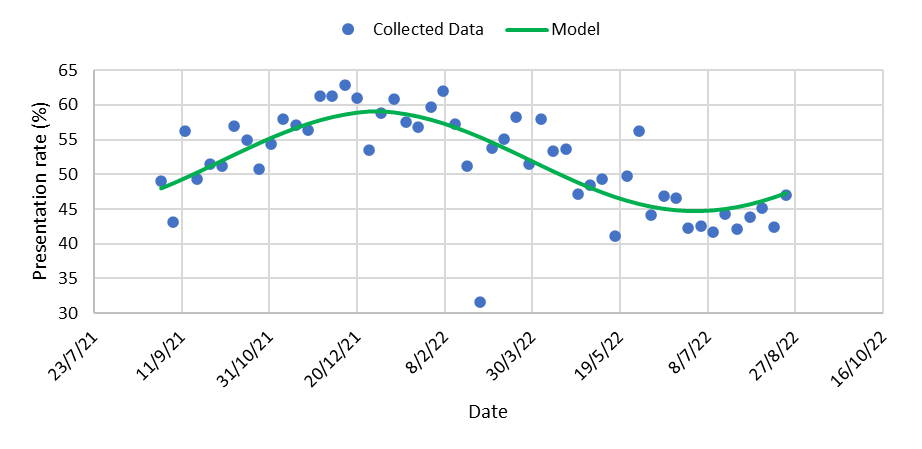


Figure S3: Presentation rate with sine wave overlaid and fitted by least squares method. R^2^≈0.5354


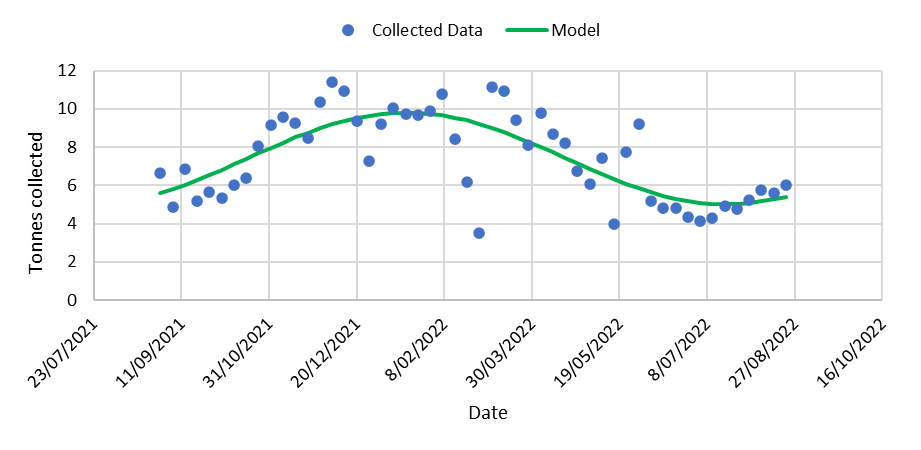


Figure S4: FOGO collection tonnage collected weekly with sine wave overlaid and fitted by least squares method. R^2^≈0.5674.


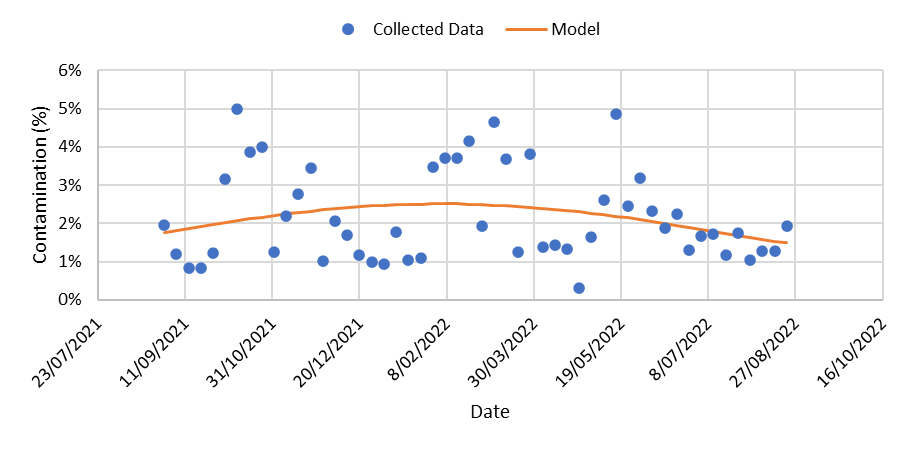


Figure S5: Contamination rate with sine wave overlaid and fitted by least squares method. R^2^≈0.0621.

# Reference List

.idcommunity (2023) *Lockyer Valley Regional Council Community Profile*. Available at: https://profile.id.com.au/lockyer-valley/population-estimate#:~:text=The%20population%20estimate%20for%20Lockyer,South%20East%20Queensland%20was%202.30%25.

Queensland Government (2022) Environmental Authority EPPR00620313. In: Science DoEa (ed).

Radicle Agriculture (2023) *HEAPS*. Available at: https://radicleag.com.au/pages/heaps.
